# Book Reviews

**Published:** 1915-07

**Authors:** 


### BOOK REVIEWS

---

Books mentioned may be inspected at and ordered through this office. So far as possible, books received in any month will be reviewed in the issue of the second month following.

---

**The Gold Fish,** published by Century Co., author anonymous.

This purports to be the analysis by himself of the life of a successful lawyer, not quite a millionaire in capital but with a millionaire's income. On some accounts we rather suspect that the book is a tract and not so literally a literary clinic. For example, while the subject confesses almost exactly the same ignorance of the personnel of political offices and of exact political methods as most men not actively engaged in politics, this is scarcely compatible with the claim that he could have immediate access to the president and senators. The author dilates on the dependence of himself, the head of the firm, on his subordinates but it is more socialistic than just and certainly more socialistic than most executives would admit, to admit that, on this account he is not earning his money by genuine service. He describes himself as generous in small ways and as giving to charities and philanthropies about as he is expected to do, yet with an expense of only $1500 in a total budget of $75,000—2%. He, of course, admits a general consignment to the waste basket of begging circulars as do most of us poorer men. In these days of organized charity, whirlwind campaigns, team work in solicitation, every-member canvasses, etc., based on carefully compiled unofficial assessment lists of the well-to-do and rich, we question seriously whether anybody could escape thus easily and maintain a reputation short of niggardliness. One little detail arouses our scepticism as to the "clinical" verity of this book, more than any other. We can imagine a man of 50, whose actual office hours are short, keeping up his business even in a whirl of social dissipation, with the help of or in spite of a rather liberal use of coffee, alcoholics, tobacco, etc. But, with European and domestic travel described as rather extensive, with an active life as a man of affairs, it seems to us a rather queer assortment of social diversions that entails an average of 100 elaborate dinners in private houses, in a year, with a corresponding return. No wonder he sighs for the simple life of the comfortably poor but why does he not follow the luxurious but relatively simple social life of most of our multimillionaires.

---

**State Board of Health, Michigan.** 41st Annual Report for the year ending June 30, 1913. Published at Lansing, Wynkopp Hallenbeck Crawford Co., State Printers.

An interesting report, somewhat late in appearance.

---

**Great Men and How They Are Produced.** Pamphlet, Casper L. Redfield, Chicago.

This is an elaborate statistic study, apparently showing that the chances of becoming eminent increase with the age of the father. An index of celebrities is appended. There can be no question but that too early marriage and reproduction tends toward a mediocre race but the conclusions reached are somewhat surprising, for example that the chances of greatness rise quite regularly and reach their maximum for sons of fathers of 60 and over. It may be questioned whether this represents the optimum for the average offspring.

---

**Iola Sanitarium,** Circular of Information and Annual Report, 1914.

This institution is the Tuberculosis Hospital for Monroe County, in many respects a pioneer, and a model of its kind. The President is Dr. John F. W. Whitbeck; the Vice-president Dr. Edwin H. Wolcott; the Secretary-Treasurer and Superintendent Dr. Montgomery E. Leary; the Resident physicians Drs. Harry J. Brayton and Eugene N. Nesbitt; the Rontgenologist Dr. Myron B. Palmer. The patients have increased from 189 in 1911 to 349, the patient-days from 12,202 to 50,295, indicating more than twice as long a stay for the average case. Of 340 cases, 129 died, 211 were discharged. Of the latter, 30 were considered arrested, 20 apparently arrested, 10 quiescent, 91 improved, 53 unimproved. A staff of examiners is distributed over the country. The report contains much further interesting information.

---

**A Manual of Diseases of Infants and Children.** By John Ruhrah, M. D., Professor of Diseases of Children, College of Physicians and Surgeons, Baltimore, Md. Fourth Edition, Thoroughly Revised. 12mo volume of 552 pages, 175 illustrations. Philadelphia and London: W. B. Saunders Company, 1915. Cloth, $2.50 net.

This work is of convenient size for reviewing the subject and for reference in emergencies yet it omits very little of real importance that is contained in far more voluminous works. The care of the new born, the anatomic and physiologic peculiarities of infancy and childhood, methods of examining children and of feeding precede the systematic discussion of practice as especially applied to children. Materia medica and therapeutics is (note that they do not say are) similarly adapted to pediatrics. Special chapters on medical inspection of school children including a synopsis of the Binet method of rating development, on directions for the care of poor children during the summer and on pediatric literature are added. We regret to confess our own mentality according to some of the tests given. For instance, a boy fifteen is supposed to be able to give the central thought of the following: ''Many opinions have been given on the value of life. Some call it good; others call it bad. It would be more just to say that it is mediocre; but on the one hand our happiness is never so great as we would have it and, on the other hand, our misfortunes are never so great as others would have them. It is this mediocrity of life which prevents it from being radically unjust.'' Well just what is the central thought? Again, the 15-year-old is supposed to distinguish the difference between the president of a republic and a king, which sounds simple till we consider the tremendous difference between the president of Switzerland and of Mexico and between the king of England and of Prussia. At ten, one is supposed to be able to repeat six figures yet it is a matter of daily experience that grown persons confuse the five figures of Federal telephone numbers or FORGET THEM in the process of dialing. Can you compose two sentences in a minute containing three such dissimilar words as Baltimore, money and river? Half of the children of ten can. At one year, ''paper is removed from candy before eating, the child having seen the wrapping.'' We have even fallen down on this test and recall a patient from whose stomach a bread label was removed by lavage. A considerable minority of adults in our experience, fall short of the ethical intelligence required of the child of ten in answer to the question, ''What would you do if you broke something belonging to some one else?''

The last chapter on pediatric literature, telling how to find information from the various medical indexes and listing the principal journals is something which might well be imitated by all authors.

---

**Diseases of the Digestive Organs.** With Special Reference to their Diagnosis and Treatment. By Charles D. Aaron, Sc.D., M. D., Professor of Gastro-enterology in the Detroit College of Medicine and Surgery; Consulting Gastro-enterologist to Harper Hospital. Octavo, 790 pages. Illustrated with 154 engravings, 48 roentgenograms and 8 colored plates. Cloth, $6.00, net. Lea & Febiger, Philadelphia.

The author has incorporated in this work much of his previous translation and extension of Schmidt's work on the faeces after test diets and of his splendid work on the stomach but has added the discussion of the intestines, oesophagus, liver, pancreas, including the more modern methods of research and treatment. The work also includes the medical phases of stomatology, proctology, a brief but highly important section on mineral waters, especially of the U. S., Rontgenology, arteriosclerosis diet and many other matters having practical and often very direct bearing on gastro-enterology in its broader modern conception. The work is well written, systematically arranged and beautifully illustrated. Based on scientific work, it is eminently practical in its applications. We take special pleasure in reviewing this masterly treatise since Dr. Aaron was a Buffalo boy, a graduate of the University of Buffalo and a student of the writer. Yet we feel that this hearty endorsement of Dr. Aaron's work is in no sense biased by personal friendship but is justified by conspicuous merit. This is the first book which has dealt systematically with the treatment of rectal diseases from the internists standpoint. In extent of scope, joint scientific and practical treatment of a broad subject, it may fairly be said that this book has no rival in the English language.

---

**A Reference Handbook of the Medical Sciences**; Embracing the entire range of Scientific and Practical Medicine and Allied Sciences. By various writers. Third Edition, Completely Revised and Rewritten. Edited by Thomas Lathrop Stedman, A. M., M. D. Complete in eight imperial quarto volumes. Volume V, 929 double column pages, illustrated by 733 engravings and 6 full-page plates in black and colors. Wm. Wood & Co., New York.

We have previously reviewed the first four volumes of this series and need only say that it represents the highest standards of efficiency for such a work and that it is invaluable for reference and for close study, each article—except for such terms as require merely definition—being a carefully prepared monograph by an expert.

---

**Pathological Technique.** Including Directions for the Performance of Autopsies and for Clinical Diagnosis by Laboratory Methods. By F. B. Mallory, M. D., Associate Professor of Pathology, Harvard Medical School; and J. H. Wright, M. D., Pathologist to the Massachusetts General Hospital. Sixth edition, revised and enlarged. Octavo of 536 pages with 174 illustrations. Philadelphia and London: W. B. Saunders Company, 1915. Cloth, $3.00.

This work is about equally divided between bacteriologic and histologic methods but it includes a lucid description of post mortem examinations, deals with the various "biologic" tests, animal parasites, and many chemic and microscopic problems of secretions, exudates, etc. In the last respect it approaches the function of works commonly described as dealing with clinical pathology. Perhaps the best general characterization of the book is to say that the authors have prepared a guide so that anyone with the requisite foundation in medical science, reasonable skill and patience, may attack any diagnostic problem presented by the dead or living body, toxicology excepted. Naturally, many other interesting lines of thought are suggested, as the preparation of museum specimens, experimental infection of animals, preparation of biologic therapeutic preparations, etc. The work is well written, carefully indexed and comprehensive.

---

**The Practical Medicine Series,** under general editorial charge of Drs. Charles L. Mix and Roger T. Vaughan of Chicago. Vol. II, 1915, General Surgery edited by Dr. John B. Murphy. The Year Book Publishers, Chicago. 602 pages, 180 illustrations, including colored and X-ray plates. $2.00. (Price for the entire series of ten volumes, $10).

This is, we believe, the largest and most ambitious book of the series ever published. The distinguished editor seems to have devoted considerable personal attention to the compilation and the book is not merely an abstract of current surgical literature but a critique of surgical progress. The review of goitre deserves special attention by the internist.

